# Assessing the artificially intelligent workplace: an ethical framework for evaluating experimental technologies in workplace settings

**DOI:** 10.1007/s43681-023-00265-w

**Published:** 2023-02-21

**Authors:** Ziagul Hosseini, Sven Nyholm, Pascale M. Le Blanc, Paul T. Y. Preenen, Evangelia Demerouti

**Affiliations:** 1 Department of Industrial Engineering and Innovation Sciences, Eindhoven University of Technology, Eindhoven, The Netherlands; 2 Philosophy, Philosophy of Science, and Religious Studies, Ludwig Maximilian University of Munich, Munich, Germany; 3 Sustainable Productivity and Employability, TNO-Netherlands Organisation for Applied Scientific Research, Leiden, The Netherlands

**Keywords:** New technologies, Ethics of technology, Social experiments, Workplaces, Meaningful work

## Abstract

Experimental technologies, including AI and robots, are revolutionising many types of work. For example, the logistics warehouse sector is witnessing a wave of new technologies, such as automated picking tools, collaborative robots and exoskeletons, affecting jobs and employees. Notably, it is not always possible to predict the effects of such new technologies, since they have inherent uncertainties and unintended consequences. Hence, their introduction into workplaces can be conceived as a social experiment. This paper aims to sketch a set of ethical guidelines for introducing experimental technologies into workplaces. It builds on Van de Poel's general framework for assessing new experimental technologies and translates that framework into a more specific context of work. We discuss its five principles: non-maleficence, beneficence, responsibility, autonomy, and justice. Each of these principles is applied to workplaces in general, and specifically to the logistics warehouse setting as a case study. A particular focus in our discussion is put on the distinctive potential harms and goods of work.

## Introduction

New technologies can make many work-related tasks simpler and more convenient. Instead of spending days in the library, for example, we can now find information within seconds. Moreover, productivity and efficiency have increased in many domains with the use of new technology. Robots, AI, advanced digital technologies and platforms, among other things, are quickly making their way into the workplace in many different sectors (e.g., health care, finance, agriculture, logistics, education) [8, 9, 14, 51]. However, technologies that make work more productive and efficient are not necessarily better from an ethical point of view; experimental technologies in the workplace can sometimes negatively impact work. For instance, technology can make work more complex and stressful due to information overload, partly by creating the feeling of always being 'on', tracked, and monitored [60]. In addition, it can create new (and unforeseen) problems for example, an exoskeleton may support the back, but wearing it on one's body for a few hours may be uncomfortable because of its weight [36]. Or, to use a more recent example, if universities start allowing students and researchers to use large language models—such as OpenAI’s ChatGPT—to produce text in educational and research settings, this may create difficulties for educators and other members of the academic workforce, for whom it will be unclear how to assess students’ assignments, as well as difficulties regarding what counts as original work and what counts as plagiarism [1]. In general, technology can have a wide range of impacts on members of the workforce. It may therefore raise moral issues, such as to what extent employees' autonomy and agency is guaranteed, how privacy rights regarding workplace surveillance are protected, and to what extent technology is used responsibly. Moreover, because we are not always able to predict the effects of new technologies in the workplace, and since there is inherent uncertainty in technological deployment, their introduction can often be classified as being "experimental" [55].

This paper aims to sketch a set of ethical guidelines for introducing new technologies into workplaces, where the uncertainties involved in the introduction of these technologies and their social effects are such that it makes sense to view this as a form of social experimentation. The paper builds on a general framework for assessing new experimental technologies proposed by Ibo van de Poel [55–57], and translates that framework into the more specific context of work. Specifically, it does so by relating Van de Poel's general framework to the distinctive potential harms and goods of work, as described by authors like Gheaus and Herzog [24], Danaher [15], and Smids et al. [52].

When we refer to 'new' technology in the work context, we refer to things such as the examples mentioned above. Sometimes the technologies are completely new (e.g., some new machine or a new form of AI that was previously not in use). Sometimes what is new may be that the level of automation in some previously existing technology has dramatically increased or that new functionalities are enabled (e.g., a truck driver switching to a highly automated truck instead of a conventional truck). In other words, a specific new technology could be faster and/or capable of doing (more) advanced tasks than was possible before, which might change the nature of the work performed by the human workers using the new technology [52] cf. [33].

In explaining and illustrating our suggestions, we will use experimental technologies in logistics warehouses as our main case study. The reason for that is that warehousing has recently witnessed a wave of new technologies, such as automated picking tools, collaborative robots, and advanced worker and warehouse management systems [8]. In fact, the quick growth of online shopping from home, most recently fuelled by the Covid-19 pandemic, is accelerating the speed with which orders are expected to be picked and shipped. To ensure uninterrupted fulfillment of orders, warehousing companies are particularly motivated to experiment with and quickly adopt new technologies.^1^

Our discussion below divides into the following sections. We start with some more general remarks about the ethics of technology as well as about recent ethical discussions of work (Sect. 2). We then introduce Van de Poel's [55] general ethical framework for the assessment of technologies that can be viewed as social experiments (Sect. 3). Having done that, we proceed to translate that framework into the work context, making use of the recent literature on what harms and benefits work might produce (Sect. 4). As we do this, we highlight how this applies to our particular case study of logistics warehouses. We end with a general discussion (Sect. 5).

## The need for moral boundaries for new technologies in the workplace

The idea of new technologies in the workplace as a form of social experimentation has not been discussed extensively in the organisational psychology and ethics literature on technology in the workplace. The literature has instead primarily focused on technological unemployment [15, 20], technological employment opportunities [17], the effects of technological developments on work characteristics such as skill variety [63], meaningful work [52, 58] and employee well-being [9]. Researchers have touched upon ethical questions such as whether robots can be good colleagues [39], and whether technology is under human control and who can be held accountable if things go wrong [28, 34, 65, 67]. Moreover, workplace surveillance (e.g., employees' communication, interaction, and productivity), and related privacy concerns, have been discussed from an ethical point of view [54, 60, 64]. These papers are all highly relevant to our discussion. But they do not explicitly conceptualise the introduction of new workplace technologies as a form of social experimentation, as we think one should do. To add to the existing literature, therefore, we think that it is important to reflect on what ethical guidelines ought to govern the introduction of new technologies into workplace settings where this can be viewed as a form of social experimentation.^2^

Let us first briefly consider how technology implementation can raise ethical issues in the work context. Notably, this is a context governed by agreements/contracts, on the basis of which people perform tasks in exchange for monetary (or other) rewards, and where clear rules can be set [15]. But it is also a context where power imbalances can lead to employees feeling pressured to do things they may not wish to do, which might not be explicitly specified in the contracts governing their employment [3]. And importantly, when new technologies are introduced into such contexts, this may change the nature of the work and affect those in the workplace in ethically significant ways [18, 20].

For example, employers that experiment with and adopt new technologies, e.g. AI-powered automated picking processes in warehouses, or automation of aeroplanes and trucks, might as a result sometimes 'de-skill' or potentially displace their workers [27, 31]. In other words, technology implementation might eliminate work, or at least certain key work tasks, for human workers [15, 20]. However, at the same time, it could create new employment opportunities in areas such as IT development, app creation, AI, and hardware manufacturing [33]. Moreover, employees being 'connected' via experimental technology may not only be deprived of the opportunity of having in-person interaction with each other, which is an essential part of our humanity, but also be prevented from helping one another to perform tasks or solve problems [8, 39]. In addition, ethical concerns might arise when experimenting with technologies might 'speed up, control, or streamline human labour, such as electronic productivity monitoring' [27, p. 6]. Other (unwanted) consequences could be that employees might experience that they do not have the power to make certain decisions, such as not having the freedom to turn off the 'automatic' option of some technology to regain full control over it, or misuse of their personal information.

These examples illustrate that in addition to the new technologies themselves, the choices made around technology implementation can also significantly impact the workplace. Engineers and designers are not always aware of the fact that they are making ethical decisions when developing new technology and neither are employers that implement new technology in their companies. Consequently, potential associated ethical issues and their (unwanted) consequences for workers often reveal themselves only after the technology has already been implemented [10, 48, 51].

Therefore, it is important to properly determine the moral boundaries for experimenting with and using new technologies in the workplace. In our view, it is preferable to have an ethical framework that can be applied by supervisors and employers not only in a reactive way but also in a proactive way. Several ethical principles have been proposed concerning how to assess and adopt new technologies (e.g., [2, 10, 59]. However, the ethical framework developed by Van de Poel is particularly relevant for our purposes. As noted above, we view the introduction of new technologies into workplace settings as a form of experimentation, and Van de Poel's framework is specifically about the ethics of experimental technologies more generally.

## Van de Poel's ethical framework

Van de Poel's [55] framework is based on the well-known *principlism approach* from medical ethics developed by Thomas Beauchamp and James Childress [7]. That view is based on four principles of *non-maleficence, beneficence, autonomy, and justice*, to which an additional principle of *responsibility* has been added by Van de Poel.^3^ He uses this ethical framework to evaluate and discuss the moral acceptability of experimental technologies in society.

A key idea in Van de Poel's [55] discussion we are particularly interested in here is the idea that introducing new technologies into society is a form of social experimentation. Importantly, new technologies often have unintended consequences, and people do not always use them in the ways engineers intended or expected. Moreover, technologies tend to have social influences on people's lives outside of the particular uses for which they were designed, since they are sometimes not designed using a human-centred approach [12]. The potential risks, benefits, and ethical problems that come with new technology will only be gradually learned after its implementation. By experimenting with technology, uncertainty can potentially be reduced through deliberate and systematic learning.

We think that these observations apply to new technologies in the workplace as much as to new technologies in general. Notably, Van de Poel himself does not specifically focus on any one type of new technology in any particular domain of life; he rather discusses social experiments with technology in general. Nevertheless, Van de Poel refers to a few specific technological experiments and their risks, such as nanoparticles in sunscreens and electromagnetic emissions from mobile phones. He discusses under which conditions experiments with such and other new technologies are morally acceptable in society.

The most important reason we have selected Van de Poel's framework for this paper is that the effects of new technology in the workplace can also be highly unpredictable, such as is the case in Amazon's warehouses to use one well-known example. Here robots were deployed with the intention to make jobs better and safer; however, it was revealed that workers had suffered serious injuries and accidents with the robots [6, 53]. Considering the fact that big companies like Amazon are constantly experimenting with new technologies, it is crucial to have clear ethical guidelines for what are in effect the social experiments in which they are deploying new technologies. Organisations need to be better equipped with respect to how to introduce and implement new technologies, and treating this as a social experiment helps to set ethical guidelines. This is a key challenge for a quickly evolving work context wherein dominant business models involve constantly seeking new levels of efficiency, productivity, and accuracy in things such as package handling and warehouse applications, and in which achieving humanised, decent work for human employees might come later in the list of priorities [40, 43].^4^ This potential trade-off between economic benefits and humanised work, combined with the experimental aspect of new technology and its uncertainties, makes it even more important to formulate an ethical framework to evaluate under which conditions new technologies can be deployed as morally acceptable as possible in warehouses and other technology-driven work contexts.

As mentioned above, Van de Poel's ethical framework consists of five principles, namely: (1) non-maleficence (i.e. avoiding causing harm), (2) beneficence (i.e. seeking to do good), (3) responsibility (i.e. distributing certain moral obligations, and making clear who is responsible for what), (4) respect for autonomy (i.e. protecting and guaranteeing human autonomy) and (5) justice (i.e. protecting vulnerable groups and avoiding exploitation). Additionally, to give this more substantive content in the context of experimental technologies, Van de Poel formulates sixteen sub-conditions, divided over these principles. Those subordinate principles are conditions that specify how to apply the five general principles to the case of social experiments with technology. Van de Poel's principles and sub-conditions are illustrated in Table 1 below:

**Table 1:** Van de Poel's ethical framework for new technologies

Non-maleficence (i.e., doing no harm)
1. Absence of other reasonable means for gaining knowledge about risks and benefits
2. Monitoring of data and risks while addressing privacy concerns
3. Possibility and willingness to adapt or stop the experiment
4. Containment of risks as far as reasonably possible
5. Consciously scaling up to avoid large-scale harm and to improve learning
6. Flexible set-up of the experiment and avoidance of lock-in of the technology
7. Avoid experiments that undermine resilience
Beneficence (i.e., doing good)
8. Reasonable to expect social benefits from the experiment
Responsibility (i.e., identifying who is accountable for following certain moral obligations)
9. Clear distribution of responsibilities for setting up, carrying out, monitoring, evaluating, adapting, and stopping of the experiment
Respect for autonomy (i.e., protecting and guaranteeing autonomy)
10. Experimental subjects are informed
11. The experiment is approved by democratically legitimised bodies
12. Experimental subjects can influence the setting up, carrying out, monitoring, evaluating, adapting, and stopping of the experiment
13. Experimental subjects can withdraw from the experiment
Respect for justice (i.e., fair distribution of benefits and burdens)
14. Vulnerable experimental subjects are either not subject to the experiment or are additionally protected or particularly profit from the experimental technology (or a combination)
15. A fair distribution of potential hazards and benefits
16. Reversibility of harm, or, if impossible, compensation for harm

## Applying the framework to the context of new technologies in workplaces 

As mentioned above, we will here translate the five principles of Van de Poel's ethical framework into ethical principles for new technologies in the workplace. As a case study, we focus on logistics warehouses. Our approach to translating Van de Poel's general framework to the ethics of technology in the workplace is to relate Van de Poel's general principles to specific ideas about the distinctive harms of work and distinctive goods of work, as discussed by authors like Gheaus & Herzog [24], Anderson [3], Danaher [15] and Smids et al. [52]. Accordingly, we focus especially on the non-maleficence and beneficence of work, i.e., particularly on the first and the second of the principles Van de Poel discusses. Our two most important questions below, therefore, concern what work-related harms should be avoided when experimenting with technologies in workplaces and what goods of work should be promoted when experimenting with technologies in workplaces.

### Non-Maleficence of work

#### Harms of work

The first principle of Van de Poel's framework emphasises that any harm should be prevented. To apply this idea to the context of work, we need to operate with some conception of what potential harms might be related to features of work for most workers (as opposed to harms not specifically related to work). One good way of approaching the question of what harms might be particularly related to work is to look at academic publications offering critical perspectives on work. We will here consider some different claims from the literature about the potential harms of work, with a particular focus on John Danaher's "reasons why you should hate your job", as presented in his 2019 book *Automation and Utopia* [15].

For example, Danaher [15] states that for many people in many sectors, work is a sometimes unjust, potentially freedom-undermining activity, with rules set by the organisation that workers in a modern corporation typically must simply follow and not question (see also [3]). As employers have control over the tasks and work schedules, employees must act within parameters that are determined by the employer(s). They must ask for permission whenever they want to do something outside those parameters. Strikingly, even though many people spend most of their time preparing for, performing, or recovering from work, only 15 percent of the workers are engaged, highly involved, and enthusiastic about their work and workplace, according to a global survey by the Gallup Institute [21]. Presumably, part of what explains this survey's finding is that people do not like being bossed around at a workplace in which they have very little freedom, which is also suggested by Parker et al. [42, 44].

Moreover, as a result of technological innovations, such as online platforms, the structure of work in contemporary society is changing. According to Danaher [15], working conditions are becoming more precarious for most workers. Instead of having permanent employment contracts, they often have less secure employment, and fewer benefits and protections. This could not only lead to an unpleasant and stressful working life, but also to increased income inequality, since economic rewards are not distributed fairly but mostly go to employers and technology suppliers [15]. An example of a digital platform is Deliveroo where people offer their services. This platform wanted to classify their employees as independent workers to avoid any duty to pay them a minimum wage or holiday pay, and they won this case [25]. This can be partly explained by technological changes being mostly ahead of legislation. For certain working conditions, roles, and values regarding employee rights, legislation may not exist, or these are not recognised yet.

New technology may also inhibit employee learning. Decisions that are made based on AI algorithms leave little room for employees to learn from mistakes and improve their work, since in most cases it is unknown how the algorithm came up with a certain decision [38]. This might lead to employees having a limited understanding of what happens in the workplace and why. Losing the skill and knowledge of making informed decisions on their own might undermine their work-related autonomy, whereas autonomy is related to positive work-related behaviour. Autonomy in the workplace is important since it enhances meaning and motivation at work, which in turn, promotes job performance, proactivity, and reduces turnover and absenteeism [8, 43, 52]. To give another example, deploying semi-autonomous robots in a real-world environment has demonstrated that these robots sometimes fail to complete their tasks by themselves. According to workplace observations by Rosenfeld et al. [50], human operators had to support robots when they could not solve a problem. This might result in a situation wherein employees have to perform both their own primary tasks and the tasks of a robot at the same time, and this implies that employees might end up with a high (cognitive) workload.

Moreover, with various tracking devices (e.g., wristbands), employers can even monitor their employees outside of work [15] since some employers ask their employees to track and share what would normally be regarded as private information, such as information about their health and sleep quality. Even if these tracking technologies are purportedly voluntary, employees may feel pressured and obliged to participate, because they do not want to stand out. Tracking and monitoring can be harmful to individuals' privacy [60]. According to a global survey among more than 250 HR leaders and employees, almost half of the employees do not trust their employers to protect their data, and these employees also worry about insecure software, a lack of transparency about how data is being protected, and whether the data is used for good or bad [30]. Furthermore, Newell and Marabelli [38] argue that surveillance might undermine employee motivation and ability to innovate.

In short, to respect the general non-maleficence principle as applied to the context of work when we introduce new technologies into the workplace, we need, among other things, to make sure that these new technologies do not (a) make people even less free at work, (b) do not significantly worsen employees’ working conditions and job security, (c) do not undermine learning and work-related autonomy, (d) do not potentially overload employees with more work than they had to do before without extra compensation, and (e) do not interfere with workers' privacy in unjustifiable ways. These are some examples of important potential harms that can be associated with work, and that the introduction of new experimental technologies needs to guard against to live up to the work-related non-maleficence principle. Having made these first suggestions about how the non-maleficence principle could be translated to the work context, let us now consider the particular case of logistics warehouses. This will also help to highlight risks of physical harms related to working with experimental technologies, which are of course also important to guard against in the ethics of experimental work technologies.

#### Potential harms of working with technologies in warehouses

Some of the leading new technologies which are being or will be adopted in today's logistics warehouses are collaborative robots, exoskeletons, wearable sensors, and virtual reality (VR)/augmented reality (AR) [8, 13]. Some of these technologies might entail risks of physical harm. For example, the malfunctioning of exoskeletons and dangerous movements of cobots in employees' vicinity could have harmful effects on employees' physical health [46], like Amazon's robots which brought products faster to employees, making them move and lift faster, without leaving much room for the workers’ muscles to rest.^5^ Accordingly, while some of these technologies have the potential to enhance logistics workers' work capacity, over-extending their use can also lead to physical harm.

Moreover, some of the above-mentioned technologies in warehouses may prompt changes in tasks, such as breaking a job into subtasks or completely taking it over from employees. Autor [5] argues that between 1980 and 2015 particularly routine work (e.g., repetitive, routine, or physical tasks) was greatly affected by technology since these tasks were easy to automate. Consequently, technology could reduce employees' skill use. Especially in logistics warehouses, this might lead to employees moving from active use of skills to mostly passive monitoring. In turn, this might make work less meaningful, and insofar as reducing the meaningfulness of work is viewed as a form of harm, making work more passive can be seen as a risk of harm [24].

Other risks prominent in the logistics work context include that surveillance technologies are frequently used to control employees' performance, which may be seen as freedom-undermining or dominating, and which could in addition have negative consequences for employee morale and job satisfaction, and mixed effects on their performance [43]. An example is picking tools that use algorithms to track, analyse, and inform workers about their performance during the picking processes (e.g., the time it takes reaching a picking location, scanning, and selecting a product, and putting it in a bin). These data might furthermore encourage companies to micromanage employees and increase their work pressure [15, 60].

### Beneficence of work

#### Benefits of work

The second main principle, beneficence, refers to the notion that one should not only avoid harm, but also proactively seek to do good and promote social benefits. While some authors primarily argue that work has many negative aspects associated with it (e.g., [15], other authors argue that work offers key opportunities for achieving many important goods, including goods “other than money”, as Anca Gheaus and Lisa Herzog [24] put it. These goods are often associated with what is thought of as “meaningful work” [8, 58], and with respect to some of those goods, the beneficence principle can be translated into conditions for enabling technologies to make work more meaningful for people.

A recent paper by Smids et al. [52] identifies five distinctive 'goods' of meaningful work, namely the following:

- pursuing a purpose
- collegiality/social relationships
- exercising skills and self-development
- self-esteem and recognition, and
- work-related autonomy.

Here is a brief explanation of these five goods of meaningful work. Firstly, work may provide employees a purpose to pursue and allow them to positively contribute to their field of work (“pursuing a purpose”). Secondly, work is a place where one has the opportunity, and for many people, the most easily accessible opportunity, to build relationships with others. Interacting and collaborating with colleagues also makes work meaningful (“collegiality/social relationships”). Thirdly, work allows employees to develop, exercise, and learn (new) skills. The process of mastering skills is very rewarding for most people [15, 24] (“exercising skills and self-development”). Fourthly, people not only work to earn money, but also to contribute to society, and to attain goods such as social recognition from others [24], which in turn has positive effects on their self-esteem (“self-esteem and recognition”). Finally, work also enables employees to shape their tasks and participate in decision-making processes, which has a positive impact on meaningful work (“autonomy”).

If we accept these suggested distinctive goods of work as being markers of meaningful work for the sake of the argument, the crucial question becomes whether, and if so how, experimental technologies might be used to promote these goods of work. In other words, workplaces that strive to live up to the principle of work-related beneficence, even as they introduce experimental technologies into the workplace, need to explore ways in which the use of those technologies could be compatible with, or directly promote, the five just-reviewed goods of meaningful work.

Importantly, the extent and opportunity to achieve these benefits could differ between sectors, types of work, and the status associated with particular jobs. As noted above, working with new technologies often changes the way people work. Experimenting with new technologies in the workplace, for this reason, might ideally allow humans to outsource tedious tasks to the technologies and instead focus on more stimulating tasks. For example, Lin et al. [31, p.21] argue that when technologies 'are particularly good at highly repetitive simple motions, the replaced human worker should be moved to positions where judgment and decisions beyond the abilities of robots are required'. In other words, when new technologies help out, assist with, and take over the ‘dull, dirty, and dangerous’ tasks, human workers can focus on more challenging and stimulating tasks, which might include tasks such as the coordination or supervision of the robots and handling problems, such as breakdowns and reparation of new technologies [52]. Accordingly, handing over tedious and repetitive tasks to technologies, and instead being given more challenging tasks, might help to give employees a stronger sense of having work that involves the pursuit of a valuable purpose.

It is less clear whether experimenting with technologies in the workplace could positively promote collegiality or social relationships at work. And so when it comes to the second key good of work identified by Smids et al. [52], a main goal for the technologically experimental workplace might simply need to be to make the use of the new technologies compatible with retaining and fostering good and collegial relationships within the workforce. It is worth mentioning, however, that some people who work with robots have been observed to form what appears to be social bonds with these robots – e.g., military personnel have been observed to become attached to bomb disposal robots – and some philosophers have begun discussing whether robots could ever be considered to be good colleagues [39]. Yet, for most types of workplaces, the main goal for the beneficent employer is likely to be to not allow the new technologies they introduce into the workplace to undermine employees' opportunities to have good relationships with their team members.

Let us next consider the third key good of work, namely skills and self-development. When employees can expand their tasks and enhance their skills as a result of the introduction of new technologies into the workplace, system performance as well as employee' well-being may very well end up being enhanced. According to Smids et al. [52], employees may be able to make significant changes in their tasks with the use of AI and robots (and other technologies). Since employees may potentially have the opportunity to modify and craft their job, their modified tasks (e.g., with more responsibilities) will have a clear purpose (to refer back to the first good of work again), and will seemingly be more meaningful.

This could then also potentially promote the fourth good of meaningful work: self-esteem and recognition in the workplace. Enabling successful work with new technologies might require higher educational attainment, the development of new social and emotional skills, enhanced creativity, and the exercise of high-level cognitive capabilities and other skills which might be hard to automate. In ideal circumstances, introducing new technologies into workplaces should – to also bring up the fifth good of work – involve experimentation that seeks to boost and provide opportunities for exercising work-related autonomy. A more minimal goal that the beneficent employer might have here, though, would simply be to avoid having experimentation with new work technologies threaten the work-related autonomy of the employees in the workplace – that is, the beneficent employer should, at least, strive to not make work any less autonomous because of the introduction of new technologies that they are experimenting with in the workplace.

#### Benefits of working with new technologies in warehouses

One of the potential benefits of the most-used new technologies in warehouses, such as cobots, could be that they can perform multiple tasks (e.g., packaging) alongside human warehouse employees, instead of displacing them. Robotic exoskeletons can facilitate human employees with upper limb movements, such as reaching, grasping, or lifting objects [46]. New technologies might also help to train or otherwise support the performance of employees. For example, Virtual Reality (VR) refers to systems where 'the input from the outside world is blocked and replaced by a system-generated input' [45, p.2]. One can potentially use VR technology to train warehouse employees to decrease their grabbing times and mistakes while order picking, or, to use another example, train warehouse truck drivers on how to avoid dangerous situations in a virtual setting. Augmented Reality (AR) adds virtual elements, items, or information in real-time to the physical world. Since order picking is one of the most important tasks in the logistics warehouses, employees can be supported with additional information for faster object location by using AR technologies to avoid errors (Cirulis & Ginters, 2013) and assist with the planning of logistics systems [47].

Thus, these above-mentioned technologies may potentially benefit employees by providing them (physical) assistance and prevent them from strain when performing physically demanding tasks [36, 68] As noted earlier, however, the overuse of these kinds of technologies might put an excessive strain on employees. Yet, if used in the right way, they could boost logistics workers' performance, while putting less strain on their muscles.^6^

### Responsibility, respect for autonomy, and justice

The last three principles of Van de Poel's framework and how they might be translated into the work context will now be discussed much more briefly since (a) our main focus in this paper is on the potential harms and goods of work and (b) doing complete justice to the last three principles in this context would require a much longer discussion than we can fit into an article of this format. Moreover, since we identify threats to work-related autonomy as a potential harm to work and protecting and boosting work-related autonomy as a key good of work, we have in effect already briefly discussed the principle of autonomy to some extent above. Additionally, some of the potential harms of work that Danaher identifies and that we have discussed above also relate to potential (in)justice issues related to work, so we have therefore also briefly addressed justice in the workplace above. Here, however, are some brief further reflections on the principles of responsibility, autonomy, and justice as they relate to the ethics of experimental technologies in the workplace.

The third principle, responsibility, states it should be clear who (e.g., the technology developer, manager, senior employee and/or project leader) is going to be responsible for what aspects of the introduction and the use of new technologies in the workplace. This may include making clear whose responsibility it is to ensure compliance with key ethical standards when experimenting or implementing new technology in the workplace. This means that the persons who bear this responsibility should, among other things, reflect on the potential negative consequences of new technologies in the workplace and also take responsibility for their decisions when doing their tasks (taking the principles of this framework into account).

A key topic that is frequently discussed within the ethics of technology more generally that is also of relevance here is the worry that some forms of advanced technologies—particularly different forms of AI, automated systems, or advanced robots—might create so-called responsibility gaps [16, 35]. That is to say, if and when tasks that were previously performed by human beings are handed over to technologies—e.g. to AI systems—and those were tasks for which the human beings in question were responsible, this might create unclarity about who is responsible for the performance of those tasks, as well as who is responsible for any good or bad outcomes that might result from the performance of the tasks in question.

This is often discussed with an eye to who, if anybody, would bear responsibility for any negative outcomes—such as harms or damages—that might be caused by autonomously operating technologies (e.g., [35]. But as Danaher and Nyholm [16] argue, in workplaces where people seek positive recognition for the good work they do or for good outcomes that are achieved, outsourcing tasks previously performed by human beings to technologies might also give rise to worries about ‘achievement gaps’. That is, if new technologies take over what were previously human tasks, there might come to be fewer opportunities for human beings to perform work that might be seen as genuine achievements that are worthy of praise.

Accordingly, experimentation with new technologies in workplaces ought—from the point of view of the third principle—to be done in a way that does not give rise to undesirable responsibility gaps of different kinds. Instead, the use of technology in the workplace ought to be done in a way that is compatible with clarity about who is responsible for what—both in the sense of responsibility for any harms or damages that might be produced in the workplace and in the sense of recognition for good outcomes that might be achieved in the workplace.

The fourth principle—respect for autonomy—refers to the ethical requirement that human subjects' autonomy and agency should be taken into consideration. As already noted, our discussion above has already covered issues related to workplace autonomy. But here we can also very briefly relate the discussion back to Van de Poel and his sub-conditions related to the principle of autonomy as he understands it. These mainly concern that human subjects are informed about the risks and benefits of the given kinds of technology, to enable them to make well-informed work-related decisions. Accordingly, Van de Poel's first sub-condition states that human subjects have the right to be informed about how new technologies (e.g., Automated Guided Vehicles, which automatically move orders from one place to another in warehouses) create risks (e.g., like the possibility of a collision). The second sub-condition states that a democratically legitimised body should approve and give consent regarding the experiment or deployment of new technology.^7^ The last two sub-conditions Van de Poel formulates state that human subjects should be able to influence any step of the experiment from the set-up to the evaluation and to withdraw from it at any time. As we see things, these general ideas about what the principle of autonomy requires with respect to experimentation with new technology carry over very naturally and seamlessly to the specific context of experimentation with new technologies in workplaces. So, here we will simply note that we endorse those general suggestions as being highly sensible also when applied to technology in the workplace in particular.

The last and fifth principle—justice—as Van de Poel [55] himself operationalises it, refers to the protection of vulnerable human subjects by a fair distribution of risks and benefits among these subjects, and the requirement that measures should be taken to protect them. This also carries over very naturally to the workplace context. For instance, in the technology-driven gig economy context, and as a result of legal systems being slow to adapt to technological change, the aforementioned platform workers are a good example of people who currently benefit less than others, who are vulnerable, and who need protection [22]. More generally, to protect employees and avoid distributive injustice in workplaces, matters such as employees' rights, obligations and working conditions clearly need to be taken into consideration when new technologies are experimented with in workplaces. Moreover, plans for how to compensate employees for any problems that may be caused for them by new technologies in the workplace should be made before the technologies are introduced. The more general topic of justice in the workplace—and how to promote it in workplaces where there is experimentation with new technologies—is a bigger topic than we can do justice to here. We aim to explore this issue further elsewhere.

In conclusion, our suggested ethical framework for experimental technologies in the context of workplaces and warehousing can be summarised with the help of Table 2, which maps our discussion in this section back onto the five general principles introduced in the previous section about Van de Poel’s framework.

**Table 2:** Ethical principles applied in the workplace and warehousing context

Non-maleficence of working with technology the ethically acceptable workplace using technological experimentation should have safeguards against, among other things
1. Employers power over employees
2. Harmful working conditions
3. Inhibiting learning skills and ability
4. High cognitive workload and less privacy
Beneficence of working with technology the ethically acceptable workplace using experimental technologies should promote opportunities for, among other things
1. Pursuing a purpose
2. Collegiality/social relationships
3. Exercising/developing (new) skills
4. Self-esteem and recognition
5. Work-related autonomy
Responsibility for working with technology in the ethically acceptable workplace should uphold ethical standards related to, among other things
1. The fair distribution of responsibilities at work, both for bad outcomes and good outcomes
Respect for autonomy the ethically acceptable workplace using experimental technologies should make sure to, among other things
1. Inform employees about technology's risks and benefits
2. Work with technology is approved by all relevantly affected parties
3. Allow employees to influence the deployment of technology
Justice the ethically acceptable workplace using experimental technologies should promote justice, shared benefits and prosperity by, among other things
1. Taking measures to protect vulnerable employees, and if harmed they will be compensated

This table is by no means exhaustive and is only intended to illustrate some of the most common and frequently occurring harms and benefits of new experimental technologies in workplaces

## Brief concluding discussion

Currently, when new technologies, such as AI and robots or different forms of automation, are being implemented in logistics warehouses and many other workplaces, the focus is usually primarily on efficiency, productivity, and cost reduction. However, like many other technology ethics researchers, we advocate the view that human factors and ethical aspects are equally important and that regulations regarding new technologies in workplaces should be enacted and enforced. We have discussed logistics warehouses in particular above, to have one key case study with the help of which to illustrate our main claims throughout the paper. But similar problems will also arise in other work contexts as well.

In general, as noted above, in translating more general principles of technology ethics to the work context specifically—such as the principles of non-maleficence and beneficence—one needs to work with existing theories of what harms and benefits work can give rise to. To illustrate how this translation can be done, we have here primarily focused on harms of work as described by Danaher [15], and goods of work as described by Smids et al. [52]. If other general theories of the potential harms and benefits of work are substituted for those we have used throughout the paper, the same overall argumentative strategy could produce slightly different results regarding what would need to be done in the introduction of experimental technologies into the workplace in order for these technologies to be non-maleficent and beneficial to workers.

In other words, it would be possible to agree with the overall approach we have taken in the main body of the paper while disagreeing with the choice of potential harms and goods of work that we have focused on as being the ones most suitable to focus on. However, we expect there to be significant overlap among different possible theories of what harms and benefits work might involve. Indeed, the goods of work presented in Smids et al.'s paper are derived from overlaps in the ethical and psychological literature about meaningful work. Moreover, the kinds of potential harms of work that Danaher discusses are also widely discussed in the literature about the ethics of work.

When it comes to implementing the types of ethical principles we have described above, two mechanisms could play a key role in practice. One is the development of legislation and concrete regulation, which could be done in a way that involves not only policymakers, but also key stakeholders such as industry leaders and technology experts, to keep up with technological changes. Another, more informal mechanism that can play a role is the emergence of social norms regarding new technologies (e.g. regarding AI, big data, robotics, VR/AR, 3D technology, sensors, digital twinning, etc.).

Importantly, however, while it can be hard to know how to do it in practice, engaging employees in the process of change and deployment of new technologies is also important, as it gives them a voice. Moreover, providing them with the opportunity to experimentally collaborate with the technology (a robot, cobot, or whatever)—in which technology is doing the monotonous, heavy tasks, and employees are performing the challenging cognitive, creative task might better enable employees working new technologies to see the potential advantages of the technologies in question.

In addition, enabling employees to improve their skills, having well-distributed tasks, and shared rewards for good results will not only help them to have a sense of control, but also 'enhances innovation-adoption, and reduces resistance to change and risk-avoiding defensive behaviours, which is not something one can use in the process of innovation' [40, p.16]. In fact, to develop critical technological citizenship (i.e., critically questioning the role and consequences of technology, participating critically in this process and taking responsibility), the views of all involved need to be considered. As we see things, technology development should not happen top-down only, but in collaboration with employees [40, 43]. The ethical principles formulated above inspired by Van de Poel's ground-breaking work on technology implementation conceived of as a form of social experimentation can guide such collaborations in fruitful and responsible ways.

## Data Availability

This is a purely theoretical paper. Accordingly, no data was used in this research.

## Footnotes

1 An example of such a company is Amazon, which has received critical attention in the media. In Amazon's warehouses, employees are, reportedly, pressured to pack hundreds of boxes per hour, while their productivity is tracked by a system that warns and fires employees due to lack of productivity without any supervisory intervention [11, 37, 62]. According to a survey by worker rights platform Organise [41], some Amazon employees even avoid going to the toilet at work since they are scared of missing their target. Such automated tracking of employees could not only increase the pressure to work quickly, but also lead to unsafe or unhealthy working conditions, as well as increased employee turnover due to feeling overworked and burnout [27]. In fact, between 2016 and 2019 the serious injury rates were 50 per cent higher at Amazon warehouses where robots were implemented than those without robots [6]. This example illustrates that new technology could have an immense impact on work dynamics and employees. This may always have been the case when introducing new technology, but in the fourth technological revolution this is happening faster than ever before.

2 Notably, philosophers of technology differ in whether they view technologies as inherently neutral tools ("the instrumental theory of technology"), whether they view technologies as inherently value-laden and as influencing the way human beings experience the world ("the mediation theory"), or whether some technologies might even come to take on the roles of moral agents and moral patients ("machine ethics"). [4, 26, 27, 29, 61]. Whatever your general view of technology might be, all sides will agree that new technology has the potential to change the work setting, which could affect employees' physical and psychosocial health, and have unintended implications and consequences for the way they work [36, 43].

3 Notably, a somewhat similar approach has been taken within general AI ethics by Floridi and Cowls [19], who also use the principlism bioethics framework with its four principles and add *explicability* in their study about socially beneficial artificial intelligence. We are cautiously sympathetic to this suggestion by Floridi and Cowls as a more general ideal within AI ethics, but focus on the principles formulated by Van de Poel here when we consider experimentation with new technologies (including AI) within workplaces in particular. [E.g., as it relates to the example described in 2.] Our main interest in this paper is in how Van de Poel’s ethical principles for social experimentation with technologies can be applied or extended to the specific context of experimentation with new technologies in the workplace. Moreover, we are not only concerned with AI technologies here, though such technologies are an important subset of the new technologies that may be experimented with in workplaces. It is an interesting question, which we plan to explore further in future work, whether the principle of explicability should be added as a guiding principle for experimentation with AI in workplace settings. This is an intuitively plausible idea, but one that we think requires further investigation that goes beyond the scope of what we do in this paper. For arguments to the effect that explicability/explainability may often be important, but not necessarily ethically required in all settings (including medical work settings), see [49] and [32].

4 Another relevant example that relates to the logistics warehouse context is experimentation with having AI-powered packaging technologies—in which the software is used for information processing and decision-making – collaborating with a human packer, during the performance of packing orders, meaning that it might be necessary to come up with ethically acceptable guidelines for what it means to have a shared responsibility with AI technologies for work-related actions.

5 Moreover, research shows that exposure to physical hazards, such as exertion, speed of work and hand-wrist posture in the work setting contributes to musculoskeletal disorder incidence [23].

6 If used correctly, technologies may also be able to benefit employees by providing them with useful and guided feedback that is customised to their performance which could be used to enhance employees' development and knowledge. For example, AI tools such as digital assistance systems with automated feedback loops, for example pick-by-light technology, could be helpful for handling complex tasks and processing information [66]. While there might be worries about surveillance and privacy issues, as discussed above, job feedback resulting from the use of wearables and devices could also potentially improve employees' skill mastery and learning (Parker & Grote, 2020). This knowledge might empower employees to engage in problem-solving and make complex decisions. Moreover, even though some tasks may be algorithmically assigned, it is recommended to provide employees with autonomy and freedom to decide what tasks to perform, determine the order of their tasks themselves and to expand their tasks.

7 In a workplace context, this might be taken to mean that the decision to adopt new technologies should not only be in the hands of technology developers and employers, but also other parties should take part in the decision making such as employees and public scrutiny (e.g. trade unions).
